# Chromosome-level haplotype-resolved genome assembly of bread wheat’s wild relative *Aegilops mutica*

**DOI:** 10.1038/s41597-025-04737-y

**Published:** 2025-03-13

**Authors:** Surbhi Grewal, Cai-yun Yang, Ksenia Krasheninnikova, Joanna Collins, Jonathan M. D. Wood, Stephen Ashling, Duncan Scholefield, Gemy G. Kaithakottil, David Swarbreck, Eric Yao, Taner Z. Sen, Ian P. King, Julie King

**Affiliations:** 1 Wheat Research Centre, School of Biosciences, University of Nottingham, Loughborough, LE12 5RD UK; 2 Wellcome Sanger Institute, Wellcome Trust Genome Campus, Hinxton, CB10 1RQ UK; 3 Earlham Institute, Norwich Research Park, Norwich, NR4 7UZ UK; 4 United States Department of Agriculture—Agricultural Research Service, Western Regional Research Center, Crop Improvement and Genetics Research Unit, 800 Buchanan St., Albany, CA 94710 USA; 5 University of California, Department of Bioengineering, Berkeley, CA 94720 USA

**Keywords:** Plant breeding, Plant genetics

## Abstract

Bread wheat (*Triticum aestivum*) is a vital staple crop, with an urgent need for increased production to help feed the world’s growing population. *Aegilops mutica* (2n = 2x = 14; T genome) is a diploid wild relative of wheat carrying valuable agronomic traits resulting in its extensive exploitation for wheat improvement. This paper reports a chromosome-scale, haplotype-resolved genome assembly of *Ae. mutica* using HiFi reads and Omni-C data. The final lengths for the curated genomes were ~4.65 Gb (haplotype 1) and 4.56 Gb (haplotype 2), featuring a contig N50 of ~4.35 Mb and ~4.60 Mb, respectively. Genome annotation predicted 96,723 gene models and repeats. In summary, the genome assembly of *Ae. mutica* provides a valuable resource for the wheat breeding community, facilitating faster and more efficient pre-breeding of wheat to enhance food security.

## Background & Summary

Food security is an increasingly pressing issue due to the growing global population, climate change, and the limitations of finite resources^1^. To address this, wheat breeders depend on new sources of genetic variation to develop high-yielding, resilient wheat varieties capable of withstanding various biotic and abiotic stresses^2^. The *Aegilops* (goatgrass) genus is one of the most promising genera harbouring diversity and beneficial alleles that can be exploited for wheat improvement^3–6^. Comprising 23 species^7^, it includes members of the primary gene pool, such as *Aegilops tauschii* (the donor of the wheat D subgenome^8,9^) and the secondary gene pool, like *Aegilops speltoides*, closely related to B subgenome donor^10^. Other species belong to the tertiary gene pool of bread wheat, offering additional potential for genetic enhancement^11^.

*Aegilops mutica* Boiss. (2n = 2x = 14) is a diploid wild relative of wheat, belonging to its secondary gene pool. Due to a debate about its phylogenetic position, *Ae. mutica* was excluded from the *Aegilops* genus for a long time and classified as *Amblyopyrum muticum* (Boiss.) Eig^7^. However, recent research has revealed that *Ae. mutica* is closely related to *Ae. speltoide*s^12,13^ and more than half of the diploid *Aegilops* species are believed to have originated from an ancient hybridization event involving *Ae. mutica*^14^. These findings support its placement in the *Aegilops* genus rather than *Amblyopyrum*.

*Ae. mutica* has been extensively utilised in pre-breeding programmes to enhance wheat’s genetic diversity^15–17^, particularly for various traits such as disease resistance^18^ and grain quality^19,20^. Advanced high-throughput genotyping tools, including chromosome-specific KASP markers^21,22^ and methods such as whole-genome skim-sequencing, have been developed to accurately detect *Ae. mutica* introgressions in a wheat background^23,24^.

The availability of long-read sequencing technologies has led to a growing number of high-quality, chromosome-scale genome assemblies for wild wheat relatives^25^ including several *Aegilops* species^26–29^. Two contig-level assemblies of the T genome of *Ae. mutica* (syn. *Am. muticum*) have been published to date^24,28^. *Ae. mutica* is an out-crossing species with a high degree of sequence heterozygosity and thus, the new fully-phased reference genome assembly of *Ae. mutica* presented here marks a significant improvement in terms of completeness, contiguity, and accuracy.

This haplotype-resolved assembly was based on Pacific Biosciences HiFi long reads, scaffolded to chromosome scale using Omni-C® data which uses a sequence-independent endonuclease for chromatin conformation capture^30^. The assembly was annotated with 96,723 gene models and repeats using a similar methodology to that used for the genome annotation of wheat wild relative *Triticum timopheevii*^25^. The chromosome-scale haplotype-resolved genome assemblies obtained in this study provide a reference for the T genome of the *Aegilops* genus. This new resource will form the basis for comparative genomics across different *Aegilops* species and will be explored to detect *Ae. mutica* introgressions in both durum and bread wheat allowing future genome-informed gene discoveries for various agronomic traits.

## Methods

### Plant material, nucleic acid extraction and sequencing

All plants were grown in a glasshouse in 2 L pots containing John Innes No. 2 soil and maintained at 18–25 °C under 16 h light and 8 h dark conditions.

Two grams of young, fresh leaf tissue (dark-treated for 48 hours) of *Ae. mutica* accession 2130012 (Germplasm Resource Unit, John Innes Centre available at https://www.seedstor.ac.uk/search-infoaccession.php?idPlant=27703) was collected in 2-ml microcentrifuge tubes and snap-frozen in liquid nitrogen. Frozen leaf tissue was ground under liquid nitrogen using a mortar and pestle and homogenised. High molecular weight (HMW) DNA was extracted using a modified Qiagen Genomic DNA extraction protocol (10.17504/protocols.io.bafmibk6)^31^ as previously described by Grewal *et al*.^25^. Solutions were transferred using wide-bore pipette tips to minimise DNA shearing. DNA concentration was measured using the Qubit 3.0 fluorometer (Invitrogen, USA) with the broad-range assay. Purity assessment was conducted using a NanoDrop spectrophotometer (Thermo Fisher Scientific, USA) by evaluating the A260nm/A280nm (expected range: 1.8–2.0) and the A260nm/A230nm (expected range: 1.8–2.2) absorbance ratios, and by comparing the NanoDrop vs. the Qubit concentration estimates, with an expected mQubit/mNanoDrop ratio close to 1:1.5^32^. The HMW DNA was sent to Novogene (UK) Company Limited for PacBio long-read sequencing. The DNA was sheared to the appropriate size range (15–20 kb) and PacBio HiFi sequencing libraries were constructed. Sequencing was performed on 9 SMRT cells of the PacBio Sequel II system in CCS mode to generate ~192.25 Gb (~41-fold coverage) of long HiFi reads with mean length 16,256 bp (Table S1).

Two Omni-C® libraries were prepared using 2 g of leaf sample (taken from the same plant used for HMW DNA extraction), at Dovetail® Genomics – Cantata Bio (California, USA) using the Omni-C® proximity ligation technology as part of the Dovetail® Omni-C® kit. As described by Wright *et al*.^33^, for each Dovetail® Omni-C® library, chromatin was fixed in place with formaldehyde within the nucleus before extraction. The cross-linked chromatin was then digested with DNAse I, repaired at chromatin ends and ligated to a biotinylated bridge adapter followed by proximity ligation of adapter containing ends. Following proximity ligation, crosslinks were reversed and the DNA was purified. Biotin not internal to ligated fragments was then removed. Library preparation was performed using NEBNext Ultra enzymes and Illumina-compatible adapters. Biotin-containing fragments were isolated using streptavidin beads, followed by PCR enrichment of each library. The libraries were sequenced on an Illumina HiSeqX platform to produce on average 800 million reads per library resulting in an approximately 50x sequence coverage (~240 Gb of 2 × 150 bp reads; Table S2).

Total RNA was extracted from seedlings at 3-leaf stage (dawn and dusk), as well as from roots, flag leaves, spikes and grains as previously described by Grewal *et al*.^25^. Flag leaves and whole spikes were collected at 7 days post-anthesis, and whole grains were collected at 15 days post-anthesis. In brief, 100 mg of ground tissue from each sample was used for RNA isolation using the RNeasy Plant Mini Kit (#74904, QIAGEN Ltd UK). The RNA was split into 2 aliquots: one for mRNA sequencing (RNA-Seq) and one for Iso-Seq^34^. Library construction and sequencing were carried out by Novogene (UK). RNA-Seq was carried out on the Illumina NovaSeq 6000 S4 platform, generating an of average 523 million reads (~79 Gb of 2 × 150 bp reads) per sample (Table S3). The second RNA aliquot from each of the six tissues was pooled into one sample and sequenced on the PacBio Sequel II system using the Iso-Seq pipeline, yielding 3.82 Gb of Iso-Seq data (Table S4) which was analysed using the PacBio Iso-Seq analysis pipeline (SMRT Link v12.0.0.177059).

### Cleaning of sequencing data

Pre-processing of sequence reads was carried out as previously described by Grewal *et al*.^25^. The HiFi sequencing read files in BAM format were converted and combined into one fastq file using bam2fastq v1.3.1 (https://github.com/jts/bam2fastq). Reads with PacBio adapters were removed using cutadapt v4.1^35^ with parameters: --error-rate = 0.1 --times = 3 --overlap = 35 --action = trim --revcomp --discard-trimmed. Omni-C reads were trimmed to remove Illumina adapters using Trimmomatic v0.39^36^ with parameters ILLUMINACLIP:TruSeq 3-PE-2.fa:2:30:10:2:keepBothReads SLIDINGWINDOW:4:20 MINLEN:40 CROP:150.

### Long-read genome assembly and scaffolding

The cleaned HiFi reads were assembled into the initial set of contigs using hifiasm (v.0.19.5-r587)^37^ in Hi-C mode producing haplotype 1 and haplotype 2 contig level assemblies. The latter had further haplotigs removed using purge_dups (v.1.2.6). The removed haplotigs were combined with haplotype 1. The Omni-C reads were mapped to the contig assembly of each haplotype following the Arima Genomics® mapping pipeline (available at https://github.com/ArimaGenomics/mapping_pipeline) and the generated bam files used as input for the scaffolder YaHS^38^ (v.1.2a.2; --e DNASE), generating more contiguous, scaffolded assemblies. The assembly files were screened for contamination using the Automated System for Cobiont and Contamination (ASCC) detection pipeline (https://github.com/sanger-tol/ascc) and the analysis files generated using the Nextflow analysis pipeline TreeVal (https://github.com/sanger-tol/treeval).

### Manual curation

Manual curation of the assembly was performed using Omni-C data following the Rapid Curation pipeline (https://gitlab.com/wtsi-grit/rapid-curation). The scaffolded haplotypes were combined in a single FASTA file and a Hi-C contact map was produced for the whole genome by mapping the Omni-C reads to the combined scaffolded assembly using PretextMap v0.1.9. The assembly was then visualised using PretextView v0.2.5 (https://github.com/sanger-tol/PretextView) where the scaffolds were individually interrogated for assembly errors indicated by the mapped Hi-C data. Any mis-joins, mis-phasing and missed joins were then corrected by manual manipulation of the map based on evidence from Hi-C interactions both within and between scaffolds as described by Howe *et al*.^39^.

Following 482 scaffold breaks and 716 joins two corrected and near fully phased haplotypes were produced and the scaffold N50 across the complete assembly was increased by an average of 17.8% to ~639.72 Mb for haplotype 1 and ~636.61 Mb for haplotype 2 (Table 1). Of the finalised assembly it was possible to assign 98.08% and 99.13% to 7 identified T genome chromosomes for haplotypes 1 and 2 respectively (the remainder were unplaced scaffolds). Chromosomes were named and orientated according to synteny with the reference genome of *Ae. tauschii*^40^. Final lengths for the curated genomes were 4,654,343,317 bp (haplotype 1) and 4,559,823,250 bp (haplotype 2) assessed using gfastats v1.3.1^41^.

**Table 1:** Summary statistics for haplotype-resolved genome assembly of *Aegilops mutica*.

Assembly characteristics	Haplotype 1	Haplotype 2
Number of scaffolds	1,031	339
Total scaffold length (bp)	4,654,343,317	4,559,823,250
Scaffold N50 (bp)	639,720,019	636,614,816
Largest scaffold (bp)	716,474,979	714,815,917
Average scaffold length (bp)	4,514,397.01	13,450,806.05
No. of contigs	2,812	1,967
Total contig length (bp)	4,653,987,281	4,559,497,650
Average contig length (bp)	1,655,045.26	2,317,995.75
Contig N50 (bp)	4,351,393	4,598,955
Largest contig (bp)	29,501,677	29,452,902
GC content (%)	47.25	47.26

There was one unlocalised scaffold on each of the chromosomes 5 T and 7 T in haplotype 1. In haplotype 2, there were four unlocalised scaffolds on chromosome 1 T, two on chromosome 2 T and one on chromosome 7 T. However, the lengths of these unlocalised scaffolds were included in the lengths of the chromosomes they were assigned to in each haplotype (Table 2) and were thus, not included in the total length of the unplaced scaffolds.

**Table 2:** Statistics of the *Aegilops mutica* chromosomes in each haplotype with annotated gene models for haplotype 1.

Haplotype	Chromosome	Length (bp)	Number of contigs	Number of gene models
1	1 T	575,865,044	230	12,075
	2 T	697,452,890	298	15,359
	3 T	716,474,979	306	15,319
	4 T	639,720,019	230	10,013
	5 T	636,536,909	274	14,063
	6 T	586,421,619	179	11,985
	7 T	713,374,731	226	16,306
	Unplaced	88,497,126	1,069	1,603
	**Total**	**4,654,343,317**	**2,812**	**96,723**
2	1 T	574,777,594	227	—
	2 T	687,901,940	277	—
	3 T	696,943,691	278	—
	4 T	629,773,018	209	—
	5 T	636,614,816	247	—
	6 T	579,256,678	170	—
	7 T	716,165,412	234	—
	Unplaced	38,390,101	325	—
	**Total**	**4,559,823,250**	**1,967**	—

### Organellar genome assembly

*De novo* assembly of the organelle genomes was carried out using the Oatk pipeline (v.4; available at https://github.com/c-zhou/oatk) with HiFi reads (k-mer size = 1001 and minimum k-mer coverage = 150) and using the angiosperms hidden Markov model (HMM) profile database^42^ for mitochondrial and chloroplast gene annotation. The circular chloroplast and mitochondrial contigs were assembled with a total size of 136,914 bp and 436,517 bp, respectively.

### Quality assessment

Quality assessments were carried out for each haplotype. Genome completeness was assessed using the Benchmarking Universal Single-Copy Orthologs (BUSCO v5.3.2)^43^ program with the poales_odb10 database. The assembly was also assessed with Merqury v1.3^44^ using a k-mer (31) database of the raw HiFi reads prepared using Meryl v1.3. The genome contiguity was evaluated by determining the LTR Assembly Index (LAI) using LTRretriever v2.9.9^45^.

Synteny between genome assemblies was evaluated using MUMmer’s (v.3.23)^46^ nucmer aligner (--mum -minmatch 100 -mincluster 500) and visualising the alignments on Dot (https://github.com/marianattestad/dot). Telomeric motifs were identified using the telo_finder.py script (https://gitlab.com/wtsi-grit/rapid-curation). The chromosome-level sequences of the two haploid genomes were also aligned using minimap2 v2.26 (-ax asm5 -n 10 -f 0.05--eqx)^47^ and SyRI v1.6.3^48^ was used to identify synteny and structural rearrangements. These were visualised using plotsr v1.1.1^49^ (-R -s 20000).

### Genome annotation

Gene models were generated from the *Ae. mutica* assembly (haplotype 1), following the same annotation pipeline as wheat wild relative *T. timopheevii*^25^, using REAT - Robust and Extendable eukaryotic Annotation Toolkit (https://github.com/EI-CoreBioinformatics/reat) in conjunction with Minos^50^. This is a genome annotation framework designed to integrate multiple sources of evidence, such as RNA-Seq alignments, transcript assemblies from Iso-Seq reads and alignment of protein sequences into a comprehensive annotation. It has been utilised in various plant genome projects, including wheat, to effectively annotate complex genomes^33,51^. A consistent gene naming standard^52^ was used to make the gene models uniquely identifiable.

#### Repeat identification

Repeat annotation, using the EI-Repeat pipeline v1.4.1 (https://github.com/EI-CoreBioinformatics/eirepeat), as described previously by Grewal *et al*.^25^, resulted in the classification of 77.01% of the assembly as repetitive sequences (Table 3).

**Table 3:** Classification of repeat annotation in *Aegilops mutica*.

	Class	number of elements	length occupied (bp)	percentage of sequence
Retrotransposons	SINEs	15,272	3,273,793	0.07
	LINEs	85,672	53,605,290	1.15
	LTRs: Copia	257,964	819,426,959	17.60
	LTRs: Gypsy	790,768	1,684,143,923	36.18
	LTRs: Unknown	738,739	222,119,274	4.78
DNA transposons	hobo-Activator	12,896	3,357,337	0.07
	Tc1-IS630-Pogo	64,769	8,885,843	0.19
	Tourist/Harbinger	28,203	8,444,080	0.18
	Other	794,895	515,890,401	11.09
Unclassified	---	672,181	265,443,787	5.70
**Total**		**3,461,359**	**3,584,590,687**	**77.01**

#### Reference guided transcriptome reconstruction

The REAT transcriptome workflow was used to derive gene models from the RNA-Seq reads (Table S3), Iso-Seq transcripts (101,674 HQ and 62 LQ isoforms; Table S4b) and Full-Length Non-Concatamer Reads (FLNC). HISAT2 v2.2.1^53^ was used to align the short reads with Iso-Seq transcripts aligned with minimap2 v2.18-r1015^47^ setting the maximum intron length to 50,000 bp and minimum intron length to 20 bp. Iso-Seq alignments with 95% coverage and 90% identity were selected. High-confidence splice junctions were identified by Portcullis v1.2.4^54^. RNA-Seq Illumina reads were assembled for each of the six tissues using StringTie2 v2.1.5^55^ and Scallop v0.10.5^56^, while FLNC reads were assembled using StringTie2 (Table S5). Gene models were derived from the RNA-Seq assemblies and Iso-Seq and FLNC alignments with Mikado^57^. Mikado was run with all Scallop, StringTie2, Iso-Seq and FLNC alignments and a second run with only Iso-Seq and FLNC alignments (Table S6).

#### Cross-species protein alignment

Protein sequences from 10 Poaceae species (Table S7) were aligned to the *Ae. mutica* assembly using the REAT Homology workflow as described previously by Grewal *et al*.^25^. Simultaneously, the same protein set was also aligned using miniprot v0.3^58^ and similarly filtered as in the REAT homology workflow. The aligned proteins from both methods were clustered into loci and a consolidated set of gene models were derived via Mikado.

#### Evidence-guided gene prediction

The evidence-guided annotation of protein coding genes was carried out using the REAT prediction workflow as described previously by Grewal *et al*.^25^. The pipeline has four main steps: (1) Transcriptome and homology-based gene models from REAT were classified based on alignments to UniProt^59^ proteins. Models predicted to contain full-length coding sequences (CDS) and meeting structural quality criteria (e.g., appropriate UTR length and a minimum CDS/cDNA ratio) are identified. A subset of gene models was then selected from the classified models and used to train the AUGUSTUS gene predictor^60^; (2) AUGUSTUS was run in both *ab initio* mode and using extrinsic evidence from the REAT pipeline (repeats, protein alignments, RNA-Seq alignments, splice junctions, and classified Mikado models). Three separate evidence-guided AUGUSTUS predictions were generated, each using different scoring priority based on evidence type. (3) Predicted AUGUSTUS models, REAT transcriptome/homology models, and additional protein and transcriptome alignments were integrated using EVidenceModeler (EVM)^61^ to produce a consensus gene set. (4) EVM-derived gene models were further processed using Mikado to incorporate UTR features and splice variants, ensuring a more comprehensive annotation.

#### Projection of gene models from *Triticum aestivum*

A reference set of hexaploid^50,62^ and tetraploid^25,63,64^ wheat gene models, derived from publicly available gene sets, were projected onto the *Ae. mutica* assembly with Liftoff v1.5.1^65^ (https://github.com/lucventurini/ei-liftover). Only Platinum, Gold, Silver and Bronze models that transferred completely, i.e., without base loss and with identical exon-intron structures, were retained.

Similarly, high confidence genes from the hexaploid wheat cv. Chinese Spring RefSeq v2.1^66^ assembly were projected onto the *Ae. mutica* genome using Liftoff, and only those fully transferred models were retained. From this set, “manually_curated” gene models (as annotated in Refseq v2.1) were specifically extracted.

#### Gene model consolidation

The final set of gene models was selected using Minos (Table 4), a pipeline that integrates protein, transcript, and expression data sets to generate a consolidated set of gene models (https://github.com/EI-CoreBioinformatics/minos). In this annotation, Minos was used to filter and merge gene models from the following sources which were generated as described above:

1. The three alternative evidence-guided Augustus gene builds.
2. Gene models derived from the REAT transcriptome runs.
3. Gene models derived from the REAT homology runs.
4. Gene models derived from the REAT prediction run, combining AUGUSTUS and EVM-Mikado.
5. Public and curated *Triticum aestivum* gene models of varying confidence levels, projected onto the *Ae. mutica* genome.
6. IWGSC Refseq v2.1 “manually_curated” models, projected onto the *Ae. mutica* genome.

**Table 4:** Summary statistics for the final structural annotation of the *Ae. mutica* genome.

Stat	Value
Number of genes	96,723
Number of transcripts	124,162
Transcripts per gene	1.28
Number of monoexonic genes	27.207
Monoexonic transcripts	27,994
Transcript mean size cDNA (bp)	1,644.52
Transcript median size cDNA (bp)	1.397
Min cDNA	96
Max cDNA	32,071
Total exons	554,231
Exons per transcript	4.46
Exon mean size (bp)	368.41
CDS mean size (bp)	283.95
Transcript mean size CDS (bp)	1,156.83
Transcript median size CDS (bp)	942
Min CDS	0
Max CDS	31,905
Intron mean size (bp)	690.45
5’UTR mean size (bp)	186.26
3’UTR mean size (bp)	292.01

Gene models were classified as biotypes protein_coding_gene, predicted_gene, and transposable_element_gene, and assigned as high or low confidence based on the criteria previously described by Grewal *et al*.^25^. A total of 38,771 high-confidence protein coding genes were annotated with an additional 40,217 genes classified as low-confidence (Table 5).

**Table 5:** Minos classified gene models.

Biotype	Confidence	Gene	Transcript
protein_coding_gene	Low	40,217	42,283
protein_coding_gene	High	38,771	63,532
transposable_element_gene	Low	10,226	10,333
predicted_gene	Low	4,368	4,429
transposable_element_gene	High	2,136	2,213
ncrna_gene	Low	1005	1372
Total		96,723	124,162

**Fig. 1:**
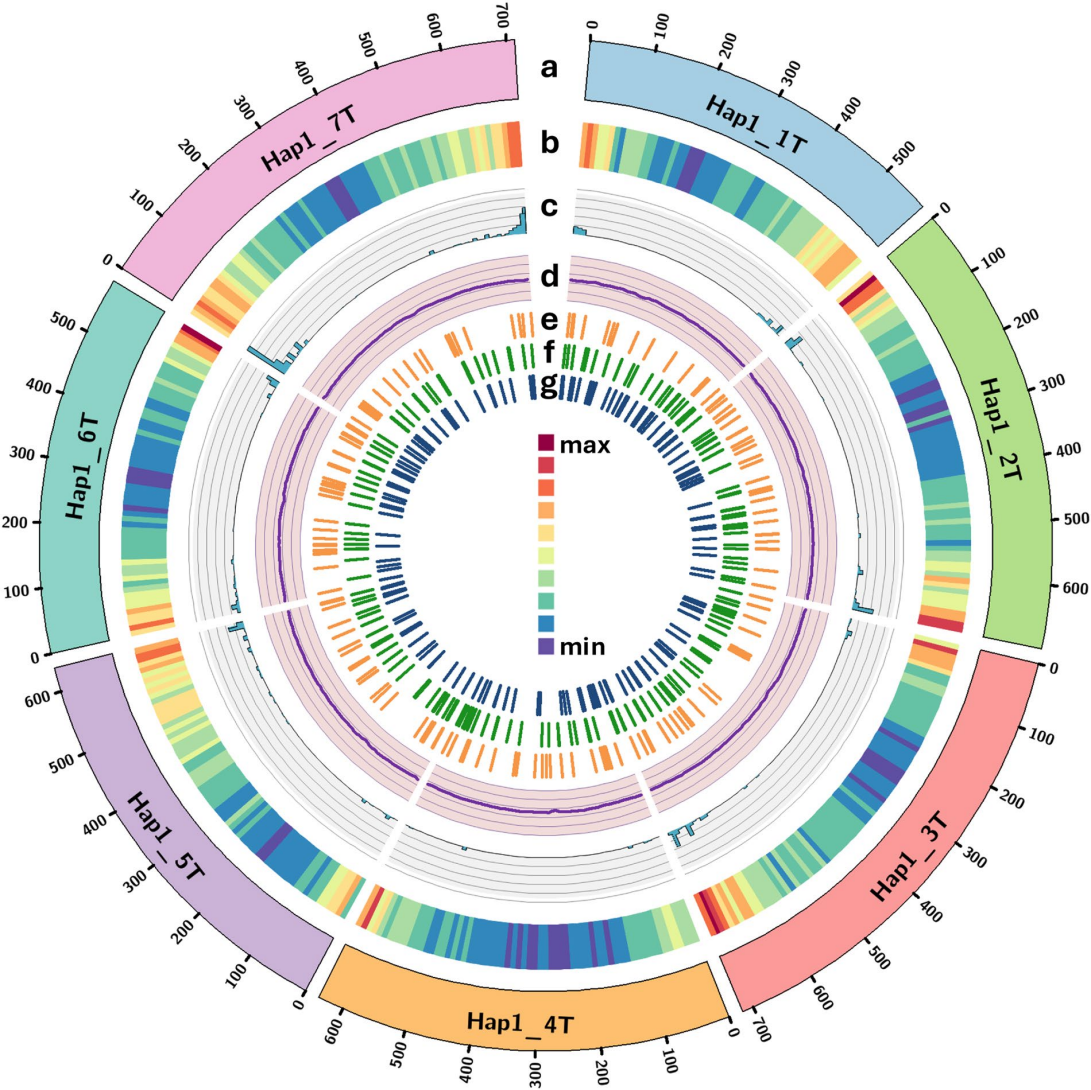
Circos plot^83^ of features of the chromosome-scale assembly of *Ae. mutica* haplotype 1 showing (**a**) T genome chromosomes (**b**) gene density (of all gene models; min = 13 and max = 665 per 10 Mb bin), (**c**) NLR density (min = 0 and max = 99 per 10 Mb bin), (**d**) GC content (in %; avg. = 47.19), and distribution of chromosome-specific KASP markers^23^ diagnostic for bread wheat’s (**e**) A subgenome, (**f**) B subgenome and (**g**) D subgenome. Y-axis for tracks c and d have an interval of 20 units.

Gene model distribution across the chromosomes and unplaced scaffolds in haplotype 1 genome is shown in Table 2 and gene density of protein coding genes and repeats across the *Ae. mutica* genome (haplotype 1) was calculated using deepStats v0.4^67^ in 10 Mb bins and shown in Fig. 1b.

#### Functional annotation

All the proteins were annotated using AHRD v.3.3.3^68^ (https://github.com/groupschoof/AHRD/blob/master/README.textile). Sequences were compared using BLAST+^69^ (blastp v2.6.0, e-value = 1e-5) against *Arabidopsis thaliana* reference proteins (TAIR10, TAIR10_pep_20101214_updated.fasta.gz - https://www.araport.org) and the UniProt viridiplantae sequences (Swiss-Prot and TrEMBL datasets download 06-May-2023). Interproscan v5.22.61^70^ results were incorporated into AHRD for functional annotation. The default AHRD example configuration file was modified as described in Grewal *et al*.^25^.

*Ae. mutica* is known as an important source for genetic variation for resistance against major diseases of wheat^18^. In total, 1060 gene models were annotated as nucleotide-binding leucine-rich repeats (NLRs) which play essential roles in plant immune systems, with a majority of cloned disease-resistance genes encoding NLRs^71,72^. The genomic distribution of these NLRs was plotted (Fig. 1c) by calculating the density in 10 Mb bins using deepStats v0.4, which shows concentration of these NLRs at mostly distal ends of the chromosomes of *Ae. mutica*.

Flanking sequence of SNPs used to design chromosome-specific KASP markers polymorphic between *Ae. mutica*^23^ and bread wheat were used in a BLAST^73^ query against the *Ae. mutica* genome (haplotype 1) sequence to determine their physical location and distribution across the *Ae. mutica* genome (Fig. 1e–g)

## Data Records

The raw sequence files for the HiFi, Omni-C, RNA-Seq and Iso-Seq reads are available at the European Nucleotide Archive (ENA) under accession number PRJEB81109^74^. The final haplotype assemblies consisting of the nuclear and organelle genomes are available from NCBI under accession numbers GCA_964657205.1 (haplotype 1)^75^ and GCA_964644865.1 (haplotype 2)^76^.

The genome assemblies, gene models, repeat and functional annotations are also available on figshare^77^.

## Technical Validation

### Assessment of genome assembly and annotation

The quality of the final haplotype assemblies was assessed via various tools (Table 6). BUSCO analysis identified 98% and 97.5% complete BUSCOs, including single-copy and duplicated BUSCOs, in haplotype 1 and 2, respectively (Table S8), indicating that the haplotype assemblies exhibited a good completeness. Merqury estimation of the consensus and completeness of the combined genome assembly indicated a consensus quality value (QV) of 65.14 and a completeness value of 95.99. The quality of the assemblies was further evaluated by determining the LTR Assembly Index (LAI) and attainment of values of 11.89 and 11.75 for haplotypes 1 and 2, respectively, suggests that the *Ae. mutica* assembly meets the criteria for a reference quality genome^45^ (LAI > 10) indicating a high level of accuracy and completeness in capturing genomic features, particularly those related to LTR retrotransposons.

**Table 6:** Assessment results of *Ae. mutica* genome completeness and quality.

Criteria	Haplotype 1	Haplotype 2	Combined
Complete BUSCOs (%)	98	97.5	—
Consensus quality value (QV)	64.65	65.71	65.14
K-mer completeness	75.96	75.29	95.99
LTR Assembly Index (LAI)	11.89	11.75	—

**Fig. 2:**
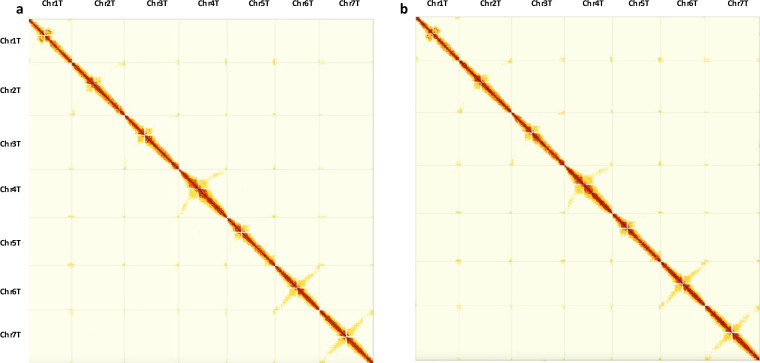
Hi-C contact maps generated by mapping Omni-C reads onto the final curated assemblies of (**a**) haplotype 1 and (**b**) haplotype 2 of *Aegilops mutica*.

The final curated haplotype assemblies were evaluated for assembly accuracy by mapping the trimmed Omni-C reads to the post-curated haplotype assemblies, as described above for scaffolding, and generating final Hi-C contact maps using PretextMap and viewed using PretextView (Fig. 2; Figs. S1–S3). Figure 2 shows a dense dark red pattern along the diagonal for both haplotypes revealing no potential mis-assemblies. To confirm the absence of phase switches, we also constructed a Hi-C contact matrix for the combined haplotype 1 + haplotype 2 assembly (Fig. S1), which supports a near fully phased genome. Additionally, zoomed-in Hi-C contact maps for each chromosome from both haplotypes (Figs. S2, S3) further validate accurate scaffolding and manual curation. The anti-diagonal patterns, (observed in some T chromosomes in Fig. 2 as well as in all chromosomes in Figs. S2, S3), are expected and have been reported for other relatively large plant genomes such as those from the Triticeae tribe^25,78^ as they correspond to the characteristic Rabl configuration of Triticeae chromosomes^79,80^.

**Fig. 3:**
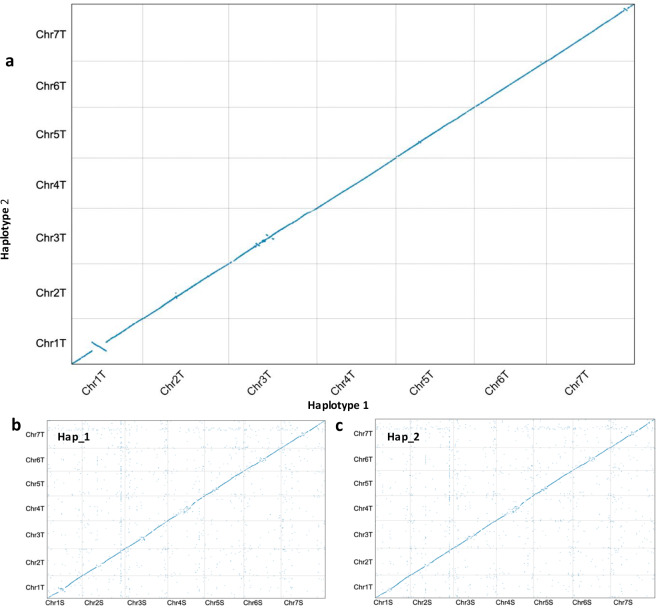
Whole-genome alignment dotplot between (**a**) the two *Ae. mutica* haplotype assemblies, (**b**) *Ae. speltoides* (S) and *Ae. mutica* haplotype 1 (T) and (**c**) *Ae. speltoides* (S) and *Ae. mutica* haplotype 2 (T).

The whole-genome alignment revealed good collinearity between the two haploid genomes (Fig. 3a) and with that of close relative *Ae. speltoides*^28^ (Fig. 3b,c). Telomeric motifs were identified at one end of 2 chromosomes in haplotype 1 (Chr 1TS and Chr3TL) and on both ends of Chr7T. In haplotype 2, all chromosomes had at least one telomere identified (Chr1TS, Chr2TS, Chr3TL, Chr4TL, Chr6TS and Chr7TS) except for Chr5T which had no motifs present (Table S9).

**Fig. 4:**
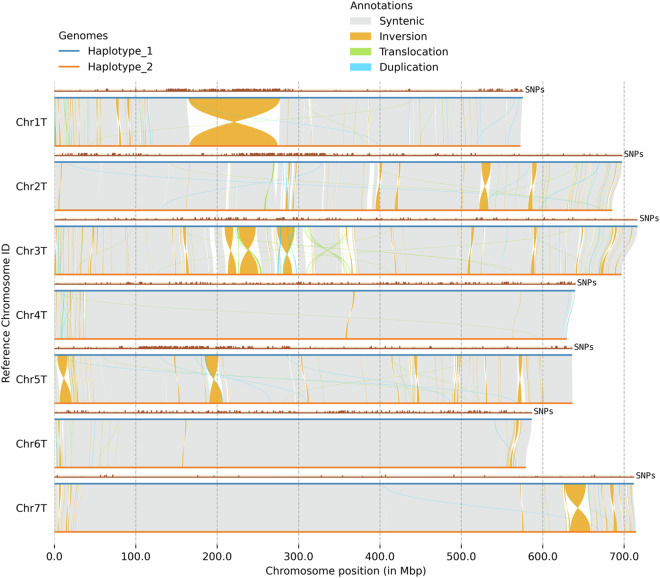
Structural variation between the two haploid genomes of *Aegilops mutica* with SNPs between the haplotypes plotted above each chromosome.

In total, 12,783 syntenic regions (approximately total 2.5 Gb) were detected (Fig. 4) between the haploid genomes. Due to the out-crossing nature of *Ae. mutica* and the resulting heterozygosity, many sequence and structural variations were discovered between the homologous chromosomes of the two haploid genomes, including 5,210,462 SNPs, 219,851 insertions, 220,040 deletions, 21,446 translocations, and 376 inversions (Fig. 4, Table S10) with largest being an inversion of ~111 Mb (166–277 Mb) near the centromere of Chr1T (Fig. 4).

Completeness of the predicted gene models was also evaluated using BUSCO and produced a score of 99.3% (0.0% fragmented and 0.7% missing BUSCOs; Table S8). The number of high confidence gene models (40,907; Table 5) is in the range of a typical diploid Triticeae species (34,000–43,000 high-confidence gene models per haploid genome)^28,81^.

## Usage Notes

A genome browser for the haplotype 1 assembly of *Ae. mutica* is currently being hosted at GrainGenes^82^ https://wheat.pw.usda.gov/jb/?data=/ggds/whe-mutica with tracks for annotated gene models and repeats and BLAST functionality available at https://wheat.pw.usda.gov/blast/.

## Supplementary information

Supplementary Information

## Data Availability

All software and pipelines were executed according to the manual and protocol of published tools. No custom code was generated for these analyses.
