# Supplementary material for: Chromosome-level haplotype-resolved genome assembly of bread wheat’s wild relative Aegilops mutica
Source: Sci Data. 2025 Mar 13;12:438. doi: 10.1038/s41597-025-04737-y (PMC11906796; doi:10.1038/s41597-025-04737-y)
Supplement: Supplementary file 1 — Supplementary Information [file 41597_2025_4737_MOESM1_ESM.docx]

**Supplemental Information**

**Chromosome-level haplotype-resolved genome assembly of bread wheat’s wild relative *Aegilops mutica***

Surbhi Grewal^1^, Cai-yun Yang^1^, Ksenia Krasheninnikova^2^, Joanna Collins^2^, Jo Wood^2^, Stephen Ashling^1^, Duncan Scholefield^1^, Gemy G. Kaithakottil^3^, David Swarbreck^3^, Eric Yao^4,5^, Taner Z. Sen^4,5^ , Ian P. King^1^ and Julie King^1^

1. Wheat Research Centre, School of Biosciences, University of Nottingham, Loughborough, LE12 5RD, UK
2. Wellcome Sanger Institute, Wellcome Trust Genome Campus, Hinxton, CB10 1RQ, UK
3. Earlham Institute, Norwich Research Park, Norwich NR4 7UZ, UK
4. University of California, Department of Bioengineering, Berkeley, CA, 94720, USA
5. United States Department of Agriculture—Agricultural Research Service, Western Regional Research Center, Crop Improvement and Genetics Research Unit, 800 Buchanan St., Albany, CA 94710, USA

Corresponding author: Surbhi Grewal ([surbhi.grewal@nottingham.ac.uk](mailto:surbhi.grewal@nottingham.ac.uk))

[Table S2. Statistics of Omni-C reads 4](#_Toc189556315)

[Table S3. Statistics of mRNA sequencing. 5](#_Toc189556316)

[Table S4. Statistics of Iso-Seq sequencing (a) and initial analysis (b) using the PacBio Iso-Seq pipeline. 6](#_Toc189556317)

[Table S5. Reference guided transcriptome assembly statistics for short read transcriptome data assembled with Stringtie and Scallop for FLNC reads assembled with StringTie. 7](#_Toc189556318)

[Table S6. REAT Transcriptome Mikado consolidated gene sets, gene model statistics. 8](#_Toc189556319)

[Table S7. List of Species used for cross species protein alignment 9](#_Toc189556320)

[Table S8. BUSCO evaluation results of (a) genome assembly of haplotype 1, (b) genome assembly of haplotype 1 and (c) gene models 10](#_Toc189556321)

[Table S9. Telomere motif sequences identified in Ae. mutica chromosomes from both haplotype assemblies. 11](#_Toc189556322)

[Table S10. Structural and sequence annotations between both haploid genomes of Ae. mutica. 12](#_Toc189556323)

[Figure S1. Hi-C contact matrix of Omni-C reads mapped onto the curated concatenated haplotype 1 and haplotype 2 assemblies showing nearly fully phased 14 chromosomes (seven T chromosomes from each haplotype) of Aegilops mutica. 13](#_Toc189556324)

[Figure S2. Zoomed in Hi-C contact maps for Aegilops mutica haplotype 1 chromosomes (a) 1T, (b) 2T, (c) 3T, (d) 4T, (e) 5T, (f) 6T and (g) 7T. 14](#_Toc189556325)

[Figure S3. Zoomed in Hi-C contact maps for Aegilops mutica haplotype 2 chromosomes (a) 1T, (b) 2T, (c) 3T, (d) 4T, (e) 5T, (f) 6T and (g) 7T. 15](#_Toc189556326)

**Table S1.** Statistics of HiFi reads.

| **BioSample** | **Cell** | **Run Accession** | **HiFi reads bases(bp)** | **Total bases(Gb)** | **HiFi reads number** | **Average HiFi reads length** | **N50** |
| --- | --- | --- | --- | --- | --- | --- | --- |
| SAMEA116132440 | S12_m64165_220612_220214 | ERR13769202 | 17,993,972,705 | 97.67 | 1,168,569 | 15,398 | 15,733 |
| SAMEA116132440 | S12_m64165_220606_085139 | ERR13769203 | 19,805,650,217 |  | 1,264,607 | 15,661 | 16,806 |
| SAMEA116132440 | S12_m64164_220424_093941 | ERR13769204 | 9,399,638,940 |  | 756,967 | 12,417 | 14,362 |
| SAMEA116132440 | S12_m64165_220614_091437 | ERR13769205 | 13,950,886,851 |  | 888,985 | 15,693 | 16,720 |
| SAMEA116132440 | S12_m64164_220616_195940 | ERR13769206 | 19,166,389,070 |  | 1,221,064 | 15,696 | 16,825 |
| SAMEA116132440 | S12_m64165_220618_015422 | ERR13769207 | 17,357,172,173 |  | 1,154,135 | 15,039 | 15,458 |
| SAMEA116132440 | A12_m64165_221030_120513 | ERR13769208 | 26,700,582,261 | 26.7 | 1,610,553 | 16,578 | 16,459 |
| SAMEA116132440 | A12_m64267e_221120_111536 | ERR13769209 | 32,160,936,637 | 67.88 | 1,904,262 | 16,888 | 16,879 |
| SAMEA116132440 | A12_m64267e_221124_233115 | ERR13769210 | 35,722,268,646 |  | 2,092,590 | 17,070 | 17,060 |
|  | **Total** |  | 192,257,497,500 | 192.25 | 12,061,732 |  |  |

# **Table S2.** Statistics of Omni-C reads

| **BioSample** | **Omni-C Library** | **Run Accession** | **Raw forward reads** | **Raw reverse reads** | **Total raw reads** | **Total No. of bases (Gb)** |
| --- | --- | --- | --- | --- | --- | --- |
| SAMEA116132440 | DTG-OmniC-385 | ERR13770169 | 403,152,682 | 403,152,682 | 806,305,364 | 120,945,804,600 |
| SAMEA116132440 | DTG-OmniC-386 | ERR13770170 | 397,538,026 | 397,538,026 | 795,076,052 | 119,261,407,800 |
|  | **Total** |  | **800,690,708** | **800,690,708** | **1,601,381,416** | **240,207,212,400** |

# **Table S3.** Statistics of mRNA sequencing.

| **BioSample** | **Tissue** | **Library_Flowcell_Lane** | **Run Accession** | **Raw reads** | **Raw data (Gb)** | **Effective(%)** | **Error (%)** | **Q20 (%)** | **Q30 (%)** | **GC (%)** |
| --- | --- | --- | --- | --- | --- | --- | --- | --- | --- | --- |
| SAMEA116132441 | Grains | EKRN230001847-1A_HMTHFDSX5_L1 | ERR13770326 | 480,849,392 | 72.1 | 97.94 | 0.03 | 97.1 | 92.93 | 52.69 |
| SAMEA116132442 | Roots | EKRN230001848-1A_HMTHFDSX5_L1 | ERR13770327 | 557,513,634 | 83.6 | 98.55 | 0.02 | 97.95 | 94.4 | 54.85 |
| SAMEA116132443 | Seedlings at dawn | EKRN230001849-1A_HMTHFDSX5_L2 | ERR13770328 | 460,823,474 | 69.1 | 98.72 | 0.02 | 98.18 | 94.95 | 56.45 |
| SAMEA116132444 | Seedlings at dusk | EKRN230001850-1A_HMTHFDSX5_L2 | ERR13770329 | 547,329,916 | 82.1 | 98.41 | 0.02 | 98.07 | 94.71 | 56.36 |
| SAMEA116132445 | Spike | EKRN230001851-1A_HMTHFDSX5_L2 | ERR13770330 | 513,462,614 | 77 | 98.55 | 0.02 | 97.96 | 94.46 | 55.44 |
| SAMEA116132446 | Flag Leaf | EKRN230001846-1A_HMTHFDSX5_L1 | ERR13770331 | 582,941,820 | 87.4 | 98.1 | 0.02 | 97.94 | 94.42 | 56.19 |
|  |  | **Total** |  | 3,142,920,850 | 471.3 |  |  |  |  |  |

# **Table S4.** Statistics of Iso-Seq sequencing (a) and initial analysis (b) using the PacBio Iso-Seq pipeline.

(a)

| **BioSample** | **Tissue** | **Cell** | **Run Accession** | **HiFi reads bases(bp)** | **Total bases(G)** | **HiFi reads number** | **Average HiFi reads length** | **N50** |
| --- | --- | --- | --- | --- | --- | --- | --- | --- |
| SAMEA116132447 | Mixed pool from Table S3 | Mut_pool_m64285e_230213_153331 | ERR13770171 | 3,824,661,890 | 3.82 | 2,229,615 | 1,715 | 1,802 |

(b)

| [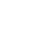](#RANGE!home)  **CCS Analysis Read Classification** | **Values** |
| --- | --- |
| Reads | 2,229,615 |
| Reads with 5' and 3' Primers | 1,837,659 |
| Non-Concatamer Reads with 5' and 3' Primers | 1,832,878 |
| Non-Concatamer Reads with 5' and 3' Primers and Poly-A Tail | 1,828,558 |
| Mean Length of Full-Length Non-Concatamer Reads | 1,679 |
| Unique Primers | 1 |
| Mean Reads per Primer | 1,837,659 |
| Max. Reads per Primer | 1,837,659 |
| Min. Reads per Primer | 1,837,659 |
| Reads without Primers | 391,956 |
| Percent Bases in Reads with Primers | 0.8416 |
| Percent Reads with Primers | 0.8242 |
| Number of High-Quality Isoforms | 101,674 |
| Number of Low-Quality Isoforms | 62 |

# **Table S5.** Reference guided transcriptome assembly statistics for short read transcriptome data assembled with Stringtie and Scallop for FLNC reads assembled with StringTie.

| **Sample_Code** | **Sample_Description** |
| --- | --- |
| Mut_Flg | Mutica Flag Leaf |
| Mut_Grn | Mutica Grains |
| Mut_RT | Mutica Roots |
| Mut_Ypl_am | Mutica seedlings at dawn |
| Mut_Ypl_pm | Mutica seedlings at dusk |
| Mut_Spk | Mutica Spike |

# **Table S6.** REAT Transcriptome Mikado consolidated gene sets, gene model statistics.

| **Stat** | **Mikado (IsoSeq and FLNC)** | **Mikado (IsoSeq + FLNC + StringTie + Scallop)** |
| --- | --- | --- |
| **Number of genes** | 23,026 | 45,264 |
| **Number of Transcripts** | 26,096 | 67,730 |
| **Transcripts per gene** | 1.13 | 1.5 |
| **Number of monoexonic genes** | 3,153 | 6,993 |
| **Monoexonic transcripts** | 3,313 | 7,679 |
| **Transcript mean size cDNA (bp)** | 1,923.95 | 1,820.71 |
| **Transcript median size cDNA (bp)** | 1,737 | 1,611 |
| **Min cDNA** | 313 | 201 |
| **Max cDNA** | 11,611 | 16,915 |
| **Total exons** | 154,299 | 357,825 |
| **Exons per transcript** | 5.91 | 5.28 |
| **Exon mean size (bp)** | 325.39 | 344.63 |
| **CDS mean size (bp)** | 252.4 | 245.53 |
| **Transcript mean size CDS (bp)** | 1,340.40 | 1,091.65 |
| **Transcript median size CDS (bp)** | 1,197 | 908 |
| **Min CDS** | 0 | 0 |
| **Max CDS** | 10,899 | 16,077 |
| **Intron mean size (bp)** | 486.22 | 543.25 |
| **5'UTR mean size (bp)** | 196.48 | 250.4 |
| **3'UTR mean size (bp)** | 338.76 | 404.01 |

# **Table S7**. List of Species used for cross species protein alignment

| **NCBI RefSeq ID** | **Species** |
| --- | --- |
| GCF_000003195.3 | *Sorghum bicolor* |
| GCF_000005505.3 | *Brachypodium distachyon* |
| GCF_000263155.2 | *Setaria italica* |
| GCF_001433935.1 | *Oryza sativa* |
| GCF_002162155.2 | *Triticum dicoccoides* |
| GCF_002211085.1 | *Panicum hallii* |
| GCF_002575655.2 | *Aegilops tauschii subsp. strangulata* |
| GCF_016808335.1 | *Panicum virgatum* |
| GCF_902167145.1 | *Zea mays* |
| GCF_904849725.1 | *Hordeum vulgare* |

# **Table S8.** BUSCO evaluation results of (a) genome assembly of haplotype 1, (b) genome assembly of haplotype 1 and (c) gene models

|  | **(a) Genome assembly of haplotype 1** | | **(b) Genome assembly of haplotype 2** | | **(c) Gene models** | |
| --- | --- | --- | --- | --- | --- | --- |
|  | Value | % | Value | % | Value | % |
| **Complete BUSCOs** | 4798 | 98 | 4773 | 98 | 4864 | 99.3 |
| **Complete and single-copy BUSCOs** | 4533 | 92.6 | 4547 | 92.9 | 4572 | 93.4 |
| **Complete and duplicated BUSCOs** | 265 | 5.4 | 226 | 4.6 | 292 | 6 |
| **Fragmented BUSCOs** | 9 | 0.2 | 13 | 0.3 | 0 | 0 |
| **Missing BUSCOs** | 89 | 1.8 | 110 | 2.2 | 32 | 0.7 |
| **Total BUSCO groups searched** | 4896 | | 4896 | | 4896 | |

# **Table S9**. Telomere motif sequences identified in *Ae. mutica* chromosomes from both haplotype assemblies.

# **Table S10.** Structural and sequence annotations between both haploid genomes of *Ae. mutica.*

**a. Structural annotations**

| **Variation_type** | **Count** | **Length_ref*** | **Length_qry^** |
| --- | --- | --- | --- |
| Syntenic regions | 12783 | 2503888862 | 2495603071 |
| Inversions | 376 | 327566950 | 317808138 |
| Translocations | 21446 | 107804529 | 108015595 |
| Duplications (reference) | 2702 | 14230264 | - |
| Duplications (query) | 35140 | - | 156843030 |
| Not aligned (reference) | 35476 | 1615301424 | - |
| Not aligned (query) | 65573 | - | 1437764426 |

**b. Sequence annotations**

| **Variation_type** | **Count** | **Length_ref** | **Length_qry** |
| --- | --- | --- | --- |
| SNPs | 5210462 | 5210462 | 5210462 |
| Insertions | 219851 | - | 22682314 |
| Deletions | 220040 | 22626832 | - |
| Copygains | 226 | - | 11597138 |
| Copylosses | 253 | 7645349 | - |
| Highly diverged | 24165 | 405772985 | 383923550 |
| Tandem repeats | 29 | 78754 | 75708 |

* ref (reference) = haplotype 1

^ qry (query) = haplotype 2


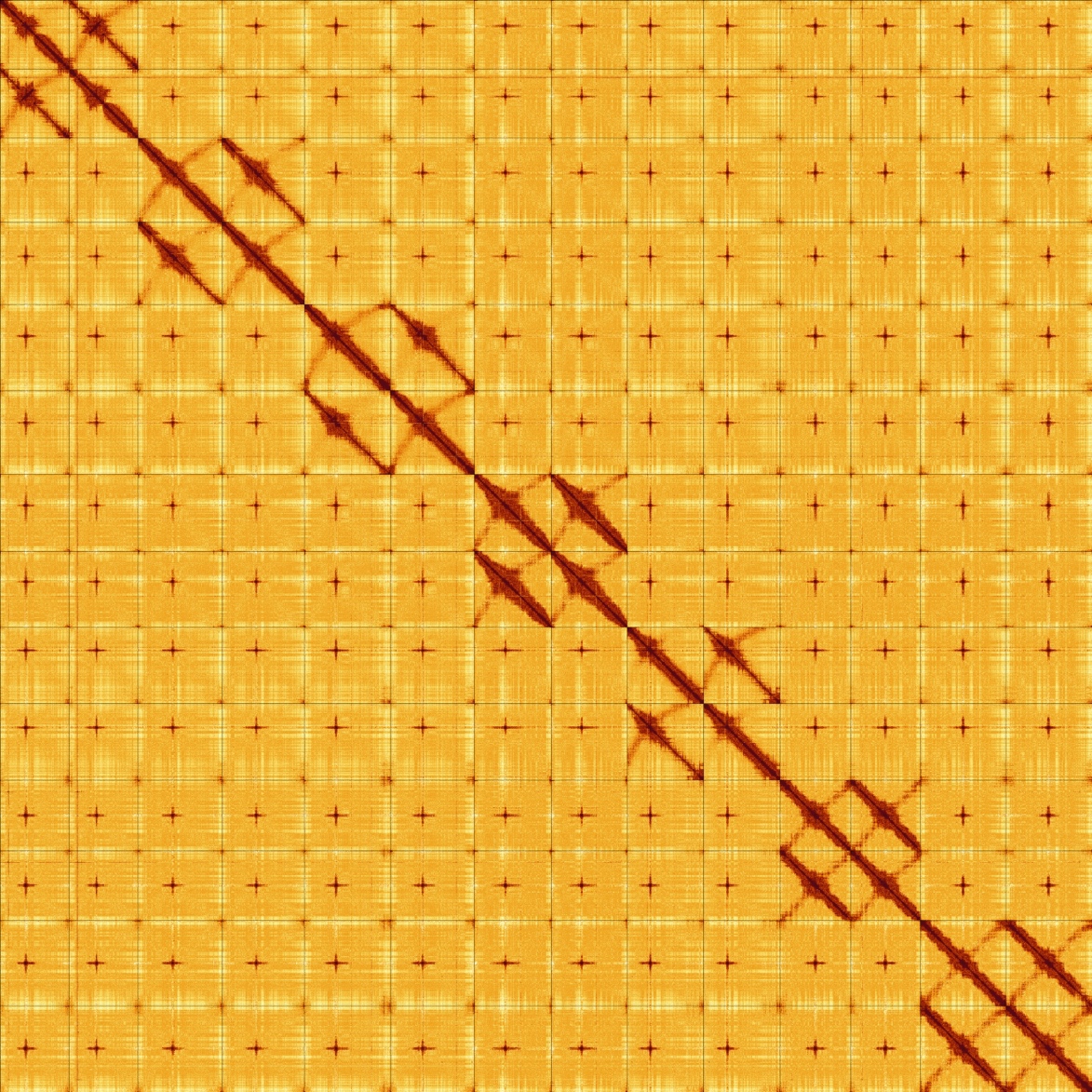


# **Figure S1**. Hi-C contact matrix of Omni-C reads mapped onto the curated concatenated haplotype 1 and haplotype 2 assemblies showing nearly fully phased 14 chromosomes (seven T chromosomes from each haplotype) of *Aegilops mutica*.


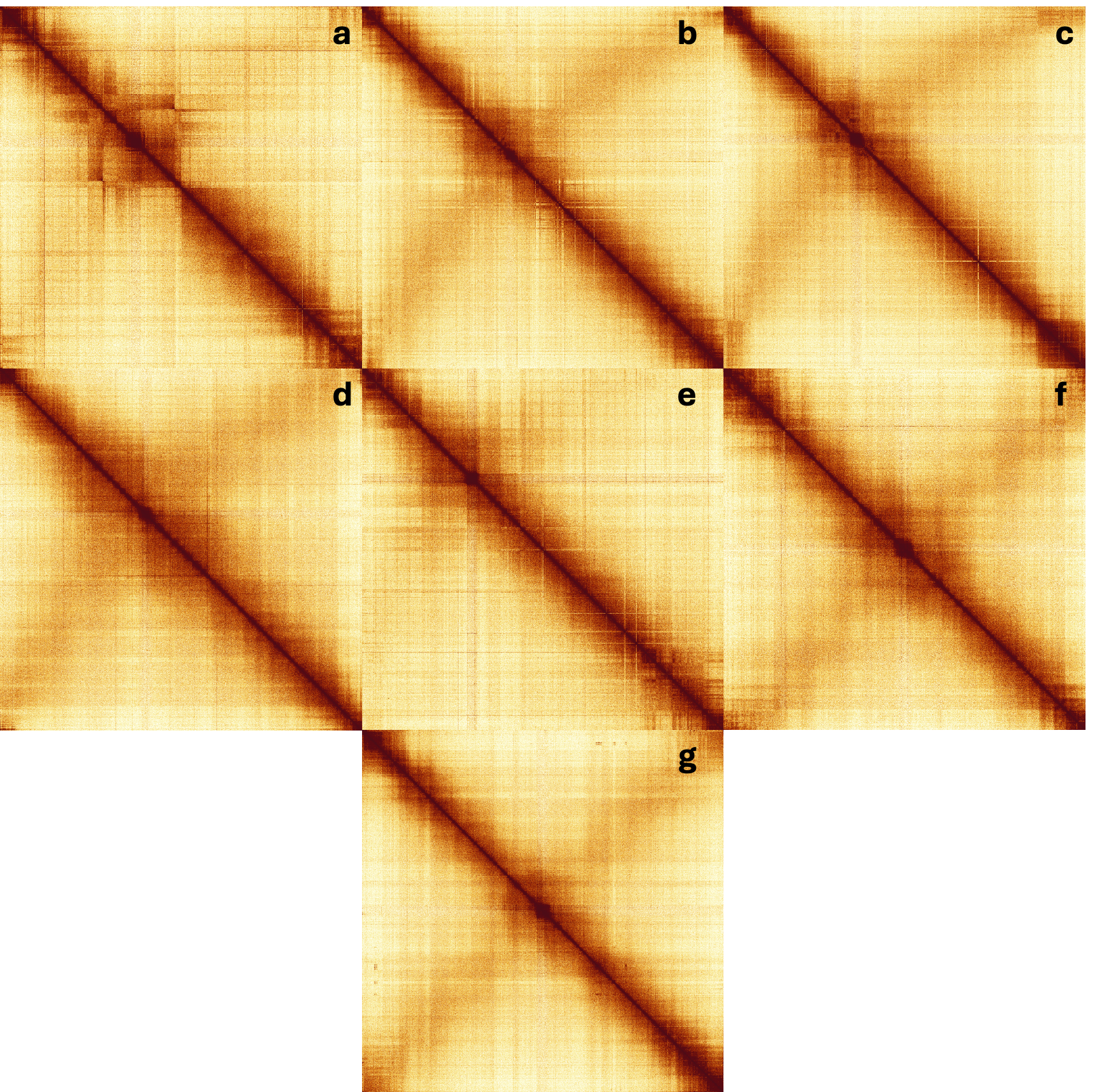


# **Figure S2**. Zoomed in Hi-C contact maps for *Aegilops mutica* haplotype 1 chromosomes **(a)** 1T, **(b)** 2T, **(c)** 3T, **(d)** 4T, **(e)** 5T, **(f)** 6T and **(g)** 7T.

# **Figure S3**. Zoomed in Hi-C contact maps for *Aegilops mutica* haplotype 2 chromosomes **(a)** 1T, **(b)** 2T, **(c)** 3T, **(d)** 4T, **(e)** 5T, **(f)** 6T and **(g)** 7T.
